# Policy evaluation of place-based strategies on upgrading county industrial structures amid economic structural transformation: Insights from pilot counties of China’s returning entrepreneurs

**DOI:** 10.1371/journal.pone.0315264

**Published:** 2024-12-27

**Authors:** Xianghui Tian, Zhang Juan

**Affiliations:** School of Economics and Management, Qingdao Agricultural University, Qingdao, Shandong Province, China; Federal University of the ABC: Universidade Federal do ABC, BRAZIL

## Abstract

In the context of the transformation of urban-rural dual economic structure, one of the important ways to realize urban-rural integrated development is to carry out county industrial structure upgrading. Based on the policy of returning home to start business as a quasi-natural experiment, this paper empirically analyzes the relationship between returning home to start business and upgrading of county industrial structure. Selecting 1997 counties across the country from 2000 to 2021 as the research sample, a multi-temporal double-difference model is used to test the impact of the place-based policy on county industrial structure and the mechanism of the impact, and the result confirms that the implementation of the pilot policy of returning home entrepreneurship plays a positive and obvious role in promoting the level of industrial development of county-level areas. Subsequent investigations reveal diverse regional impacts, notably in Central and Western and the significantly older regions; thus, the influence of the prototype program of reintroducing home entrepreneurship on enhancing industrial development is more pronounced. Viewing from a mechanistic standpoint, the pilot initiative, which motivates entrepreneurs to go back and establish new businesses, aids in enhancing industrial frameworks by boosting innovation and stimulating the market. The article offers valuable direction and understanding to decision-makers aiming to enhance the combined growth of urban and rural sectors, as well as to elevate and reform the economy.

## 1 Introduction

Amidst the city’s and rural areas’ economic overhaul, enhancing the infrastructure of county industries becomes crucial. For a long time, China’s urban and rural development has been unbalanced, and the county economy is often limited by resource conditions and market environment, with a relatively backward industrial structure. With the process of urbanization and economic development, counties are faced with the urgent need to accelerate industrial upgrading. China has implemented various local policies to enhance economic frameworks for urban and rural areas. Beginning in 2015, the National Development and Reform Commission(NDRC) along with allied organizations has orchestrated 341 pilot counties, including cities and districts, divided into three groups, to foster rural entrepreneurship and aid rural migrant workers’ repatriation to their origins. Considering the shift in the economic duality of urban versus rural regions, can this policy elevate the industrial setup of counties, identify its operational mechanism, and explore potential variances?

Academics, both domestic and international, hold varied opinions regarding policies related to locations [1]. On the one hand, according to the thinking principle of "policy facts—empirical evidence", there are direct and indirect impacts of place-based policies, where the direct impact refers to the measures, policies and mechanisms used by the government to create direct or indirect external resources for policy pilots; while the indirect impact refers to the government’s policy to promote and strengthen the formation of industrial agglomeration [2], and to improve the efficiency of resource utilization through economies of scale, innovation spillovers, market competition mechanisms and so on. The indirect impact refers to the government policies to promote and strengthen the formation of industrial agglomeration [2], and improve the efficiency of resource utilization through economies of scale, innovation spillovers and market competition mechanisms. In recent years, the Chinese government has implemented a series of place-based policies, which have confirmed the positive impact of place-based policies on economic growth [1, 3, 4], industrial upgrading [5], innovation development [6–8], and environmental improvement [9, 10]. On the other hand, from an efficiency perspective, redesigning the spatial allocation of capital and labor without fully considering the broader regional and local context can lead to a decline in productivity at the national level, a reduction in aggregate returns, and "ineffective" place-based policies [1, 11]. In developing and less developed countries, government policies to increase infrastructure investment in peripheral regions [12–14] and to support declining industries [15] often lead to greater economic agglomeration and regional polarization because countries are too small or geographically dispersed. Peripheral economies become increasingly marginalized and unable to sustain the agglomeration and domestic market effects necessary for economic growth [16], often ending up with "wasteful strategies" [17, 18].

In summary, the influence of place-based policies on regional development is multifaceted and varied. While such policies can have beneficial effects, they also carry the risk of exacerbating regional disparities. Therefore, this paper examines whether industrial structure can be influenced by location policy under the background conditions of dual economic structure transition by building a multi-temporal double difference model. The main marginal contributions of this paper, relative to the established literature, include:(1) This paper, using the pilot policy of returning to the hometown for entrepreneurship as an evaluating object provides a reference for assessing the effectiveness of place-based policies and is a good complement to the existing literature on place-based policy research. (2) A basic examination reveals a difference between areas with high and low population density, assessing whether pilot measures have lessened the industrial stagnation due to an aging populace. (3) Based on the push (technological innovation)-pull (market demand) mechanism brought about by the policy of returning to the hometown to start a business, the economic benefits of the government policy of returning to the hometown to start a business are deepened and expanded, which provides a more comprehensive reference basis for the formulation of government policy. This paper proposes the following hypotheses:

H1: The pilot policy of returning home to start a business is conducive to promoting the optimization of rural industrial structure.
H2: In the severely aging area, the pilot policy of returning home to start a business is more conducive to promoting the upgrading of industrial structure.
H3: The initial strategy of repatriating for entrepreneurial purposes can enhance the industrial framework via the effect of fostering innovation.
H4: The pilot policy of returning home to start a business can promote the upgrading of industrial structure through the market pull effect.

## 2 Distinctive facts

### 2.1 Background of the policy on entrepreneurship

After the 20th century, China’s circulation system gradually relaxed, rapid population flow. With the increase of urban labor force and the decrease of labor demand in labor-intensive industries and enterprises, the transfer of rural labor force for employment faced a severe situation. Declining expected returns and decreasing relative returns are considered to be the main factors of this phenomenon, while another reason is that a considerable proportion of rural migrant workers who move from the countryside to the city to work have not been fully integrated into the local social and living environment.

In the context of ‘mass innovation and entrepreneurship’, the number of entrepreneurs returning to their hometowns has been growing rapidly since the 1990s, when working people began to return to their hometowns to start their own businesses, as well as to cope with the huge impact of public health emergencies on the employment of rural migrant workers. According to the data of the "2022 Rural Migrant Worker Monitoring Survey Report", the total number of migrant workers in the country in 2022 is 295. 62 million. Among them, the growth rate of local rural migrant workers is faster than that of those who go out for employment. On the one hand, the scale of rural migrant workers moving to cities and towns for employment is huge, and a large number of returnees have chosen to return to the countryside to engage in innovative and entrepreneurial activities, which in turn have made positive contributions to the development of modern agriculture and the construction of new rural areas. This trend has shown a favorable development trend in terms of a gradual increase in the number of people, a wide range of fields involved, and a gradual increase in the starting point. They play an important and unique role in promoting rural economic development and are a new agricultural force. However, clear paradoxes and issues obstruct the reentry and entrepreneurial growth of migrant workers. The natural susceptibility of emergent enterprises, coupled with the scarcity of chances for assimilation into local societies and a shortage of suitable competencies, influence employees’ choices to go back to their homeland.

### 2.2 Policy tools and support measures

The Returning Entrepreneurship Policy adopts a multi-level support system covering key areas such as capital, taxation, land and human resources to ensure that returning entrepreneurs can successfully enter the market and achieve long-term development.

The State has deepened the ‘release of administrative services’ reform and further optimized the business environment for returning to rural entrepreneurship. This initiative aims to enhance governmental decentralization, upgrade entrepreneurship service systems, and foster maturity. Furthermore, enhance fiscal and tax advantages to slash the production and operational costs for entrepreneurs reopening or setting up their ventures in their hometowns. Propose creative ways for monetary support and reduce or abolish taxes and fees. Furthermore, there’s an imperative to innovate financial services to overcome the monetary difficulties of entrepreneurs staging a comeback. Originating from improved credit support tactics, it strongly encourages direct investment and explores innovative avenues. Ensure structures are simultaneously expanding collateral options to encourage economic evolution. Fourth, improve policies that bolster land, aiming to expand productivity and operational zones for returning entrepreneurs. Improve techniques for using land to maximize the value of real estate. Fifth, improve human resource effectiveness to accelerate the growth of both repeat and newly-emerged entrepreneurs. We are dedicated to enhancing local skill training, accelerating the advancement and dissemination of vocational training platforms, and actively attracting skilled individuals. For example, Xuzhou District in Yibin City, Sichuan Province, has set up an innovative ‘1+N’ service platform for entrepreneurship in the hometowns, which provides remote online services for people returning to their hometowns. Sixthly, the enhancement of applicable support centers and services will be undertaken to bolster foundational support skills for entrepreneurship and rural growth in returning home. For example, Dexing City in Jiangxi Province has invested 800 million yuan to build a demonstration base for returning to the hometown to start businesses covering an area of 505 acres, and has constructed more than 400,000 square meters of standard workshops and supporting facilities; Ruzhou City in Henan Province has constructed the RuXiu Rural Migrant Workers Returning to the Hometown to Start a Business Industrial Park covering an area of more than 4,000 acres, and has implemented a three-year rent-free policy. The suggestion is to enhance the coordination of work and policies for those returning to rural areas to initiate businesses via organized efforts within 3–5 years, enhance the policy framework for re-entering rural areas to initiate businesses, refine the setting for such re-entry, harness the zeal of major market organizations, and bolster the efforts of t shifting industrial activities to rural areas to boost job opportunities there.

### 2.3 The basic logic of the pilot policy on entrepreneurship

First, industrial orientation: integration of agriculture and modern technology. The policy encourages returning entrepreneurs to develop industries that combine with local resources and industrial advantages, such as speciality agriculture, rural tourism, rural e-commerce, etc. It also supports the development of high-tech industries and modern service industries, so as to promote the optimization and upgrading of industrial structure.

Second, the basis of reality: e-commerce and industrial chain collaboration. It supports returnees to sell agricultural products through the Internet and cooperate with live e-commerce and social media platforms to brand and market rural products. The policy encourages entrepreneurs to focus on the layout of the local characteristics of the industrial chain, supplementing the supply of upstream raw materials or extending the downstream product market, forming a ‘complementary chain, strong chain, chain extension’ mode, and enhancing the competitiveness of industrial clusters.

Third, agglomeration theory: the externalities of industrial agglomeration. The policy encourages returning entrepreneurial projects to develop in the direction of agglomeration and industrialization, and improves the success rate and collaboration capacity of entrepreneurial projects. Local governments set up business incubators, crowdsource spaces and model industrial parks to provide low-cost office space and technical support for entrepreneurs. Developed regions in the east and less developed regions in the centre and west have strengthened their cooperation to promote the transfer of industries and technologies and jointly develop the economy of entrepreneurship in the hometowns.

### 2.4 Effectiveness of the policy on entrepreneurship

By continuously improving the policy system and optimizing the supportive environment and public services, the policy of returning to the countryside to start a business will not only contribute to the revitalization of the rural economy, but will also promote the coordinated development of urban and rural areas. In the future, with the gradual improvement of policies around the world and the release of the vigor of market players, entrepreneurship in the countryside will inject more new kinetic energy into the rural economy and provide sustained impetus for the construction of modern agriculture and the countryside. According to the 2022 China Rural Revitalization Industry Integration Development Report, by 2020, the number of entrepreneurial and innovative people returning to their hometowns and villages will reach about 10.1 million nationwide, with more than 85 per cent of the entrepreneurial projects belonging to the type of integration of primary, secondary and tertiary industries, and more than 60 per cent of the projects having innovative elements. According to the monitoring of the Ministry of Agriculture and Rural Development, as of June 2021, 87 per cent of the business premises of entrepreneurship and innovation projects for returning to the hometowns and villages were set up in townships and villages and below, 70 per cent of them had the effect of driving farmers to increase their income through employment, and 40 per cent of them drove farm households out of poverty.

## 3 Theoretical analysis

The upgrading of the county’s industrial structure is mainly reflected in the optimization and upgrading of the supply and demand sides. The self-promoting ability of the county economy seems to be relatively insufficient in promoting the upgrading of industrial structure, and its relatively low industrial level, insufficient supporting facilities and relatively low entrepreneurial activity are highlighted. New economic geography suggests that peripheral regions have lower entrepreneurial activity in most industries and lower entrepreneurial intensity than core regions [19]. Although there is growing evidence of entrepreneurial activity in rural areas, entrepreneurship is primarily an urban event [20, 21]. Some studies have shown that returning entrepreneurship is influenced by personal characteristics, savings accumulation, human social capital and time spent outside [22], while the pilot policy of returning entrepreneurship promotes the flow of factors between cities and villages, and returning entrepreneurs bring market awareness, technology and capital needed by the county’s economy. Consequently, the focus of this document is on the impact of fostering innovation and the influence of market forces, in that order.

(1) Innovation promotion effect. Entrepreneurship plays an important role in a country’s innovation and economic growth [20, 23, 24], and technological innovation of enterprises can promote industrial structure upgrading [25, 26]. According to the Bower effect, the productivity differences caused by technological progress in different industries, which in turn affect the relative prices of products, ultimately stimulate the supply-side drive of industrial structure upgrading [27]. Returning entrepreneurs bring advanced technologies and management methods from cities to their hometowns, enhancing local productivity and quality. Returnees who have been away from the county for an extended period of time may be exposed to more advanced and cutting-edge technology and management concepts in the cities. This promotes the upgrading of the industrial structure toward higher value-added and more innovation. At the same time, the success stories of entrepreneurs returning to their hometowns can often effectively stimulate the entrepreneurial enthusiasm of local residents, creating the so-called "herd effect" and "demonstration effect". On the other hand, a part of the returnees will return to the city after encountering setbacks, and they will transform the setback manager into the motivation and lessons learnt for entrepreneurship; they will choose the entrepreneurial projects more carefully, focusing on the risk control and the fit of the market demand, so as to improve the probability of entrepreneurial success, and thus better promote the upgrading of the industrial structure. Therefore, by relying on the well-equipped business incubation and training bases and the rich local resource environment, large-scale returnee entrepreneurs can promote industrial agglomeration and provide industrial clusters with sufficient conditions for innovative enterprises, such as human resources, capital, and knowledge, thus forming a virtuous circle and promoting the continuous inflow of entrepreneurial resources into rural areas [28, 29].

(2) Market pull effect. Innovative entrepreneurship to optimize the supply effect as a means to more effectively meet consumer demand, thereby stimulating the development of consumption upgrading. Monitoring figures from the Ministry of Agriculture and Rural Affairs indicate that in 2020, over 85% of entrepreneurial endeavors going back to their native towns will achieve a level of amalgamation among the three sectors, with production focusing on processing, marketing, service, agriculture, culture, tourism, and education, this involves the effective development of new business models, thriving local markets, and fostering the diversification of products [30]. Exposure to new products and services leads local residents to seek higher quality, differentiated, and personalized goods. This trend expands the market for high-end products and optimizes the consumption structure. At the same time, entrepreneurial activities have generated employment, increased farmers’ income and simultaneously raised consumption levels. A rise in China’s consumption figures has been crucial in advancing the nation’s industrial framework. Returning entrepreneurs have higher consumption level and consumption demand [24]. The pilot policy of returning to their hometowns to start their own businesses promotes large-scale entrepreneurship among returnees, which has a strong pulling effect on local consumption, thus triggering the optimization of intra-enterprise allocation, alleviating overcapacity and optimizing resource allocation, and ultimately promoting the upgrading of industrial structure [31]. The expansion of consumer demand also triggers a significant "feedback effect" through the positive spatial spillover effect to strengthen the close links between industries, thus promoting the transformation and upgrading of the industrial structure of neighboring regions.

## 4 Research design

### 4.1 Model construction

This paper takes the policy of "returning to the hometown to start a business" implemented by the National Development and Reform Commission and other departments as the object of study, and aims to accurately assess the effect of the policy by using a multi-temporal double-difference model, so as to accurately identify whether the locally based policy is able to promote the upgrading of the county’s industrial structure. The quantitative analysis of this study can provide a scientific basis for government decision-making, further optimize policies, and promote the sustainable development of the county economy with the following model:

$$upgrade_{i,\;t}=\alpha_{0}+\alpha_{1}DID_{i,\;t}+\alpha_{2}X_{i,\;t}+\mu_{i}+\delta_{t}+\varepsilon_{i,\;t}$$ (1)

*upgrade*_*i*, *t*_ is the level of industrial structure in county I in year t, *DID*_*i*, *t*_ indicates whether it is a pilot county for returning entrepreneurs, and when county i becomes a pilot county in year t, it takes the value of 1 for that year and subsequent years, and 0 otherwise, *X*_*i*, *t*_ as control variables, including other factors affecting the upgrading of industrial structure, *μ*_*i*_ is a county fixed effect, *δ*_*t*_ is a year fixed effect, and *ε*_*i*, *t*_ is a randomized perturbation term.

### 4.2 Description of variables

#### (1) Explained variable: Industrial structure level

This study refers to the method of Xu Min and Jiang Yong, and applies the industrial structure upgrading coefficient to evaluate the explanatory variables. This coefficient can more intuitively reflect the development process of industrial structure from low level to high level. Through a thorough analysis of various industries’ evolution, the coefficient can precisely mirror the present industrial structure’s stage and trajectory, offering a scientific foundation and direction for the formulation and execution of industrial strategies. The increase in the value of the industrial structure upgrading coefficient reflects the degree of improvement in the level of regional industrial structure, and its specific calculation formula is expressed as follows:

$$upgrade=\Sigma_{f=1}^{3}q_{f}\times f=q_{1}\times 1+q_{2}\times 2+q_{3}\times 3$$ (2)

In the formula, *q*_*f*_ indicates the ratio of primary, secondary and tertiary industries to the total output value of the county. Values range from 1 to 3, with higher values indicating a more advanced industrial structure.

#### (2) Core explanatory variable: Pilot policies for migrant workers returning to their hometowns to start businesses

The Difference-in-Differences (DID) pilot initiative utilizes a compilation of past yearly pilot schemes for workers returning to their native towns for entrepreneurial activities.

#### (3) Control variables

It controls for important variables that affect the upgrading of the county’s industrial structure, mainly including (1) the level of economic development, measured by the logarithm of GDP per capita as an indicator. (2) Educational attainment is gauged by the proportion of students in general secondary schools to the year’s total population; (3) the level of communication, measured by the ratio of the number of fixed telephone users to the total population at the end of the year; and (4) Social welfare level, measured by the ratio of the number of beds in various social welfare institutions to the total population at the end of the year. (5) Size of government, measured by the ratio of local general government expenditure to GDP.

#### (4) Mechanism variables

The theoretical analysis in the previous section shows that the pilot policy of returning workers to their hometowns for entrepreneurship promotes the upgrading of industrial structure through the innovation promotion effect and the market pull effect, so the innovation level and the market demand level are constructed as mechanism variables. The innovation level is measured by the innovation index of Ruan Jianqing et al. 2023 "China Village Innovation and Entrepreneurship Index Report" [32]; the market demand level is measured by the logarithm of the ratio of the total retail sales of consumer goods of 10,000 yuan to the total population of 10,000 people at the end of the year.

The specific definitions of each variable are shown in Table 1.

**Table 1:** Meaning of variables.

Variable category	Variable names	Calculation method
Explained Variables	Industrial structure level	Calculate the coefficient of upgrading industrial structure according to Eq (2)
Core Explanatory Variable	Pilot policy on entrepreneurship for migrant workers returning to their hometowns	Dummy variable (0, 1)
Control Variables	Level of economic development	Lngdp
	Level of education	Number of students enrolled in general secondary schools/total population at the end of the year
	Level of communication	Number of fixed-line telephone subscribers/total population
	Level of social welfare	Number of beds in social welfare institutions/ Total population at the end of the year
	Size of Government	General budget expenditures of local finances / GDP
Mechanism Variable	Innovation level	Innovation Index of the China Rural Innovation and Entrepreneurship Index Report 2023
	Level of market demand	Total retail sales of consumer goods / Total population at the end of the year, in logarithms

The data used in this paper are panel data for 1997 counties (cities and districts) in China from 2000 to 2021. Considering the special status of municipalities and the balance of the sample, four municipalities, namely Beijing, Shanghai, Tianjin and Chongqing, are excluded. The data are mainly from the China County Statistical Yearbook, the website of the National Development and Reform Commission, the website of the Ministry of Agriculture, the website of the Ministry of Commerce of the People’s Republic of China, and the Seventh Population Census Bulletin. Table 2 presents the descriptive statistics of the variables.

**Table 2:** Descriptive statistics of the variables.

Variable names	Mean	SD	Min	Max
Industrial structure level	2.1497	0.2170	0.6656	5.8492
Pilot policy on entrepreneurship for migrant workers returning to their hometowns	0.0294	0.1690	0	1
Level of economic development	9.7020	1.0278	6.0913	12.8040
Level of education	0.0539	0.01946	0.0002	0.9042
Level of communication	0.1343	0.1281	0.0001	12.0286
Level of social welfare	21.7624	23.7592	0.0263	477.6250
Size of Government	0.2352	0.2746	0.0050	16.7352

## 5 Empirical results and analysis

### 5.1 Analysis of the baseline regression results

Table 3 shows the regression results of the multi-temporal DID model of the pilot policy of returning migrant workers to their hometowns and upgrading the county industrial structure. Columns (1) to (4) show the regression results of gradually introducing control variables, which show that each model is significant as a whole. Table 3 (1) presents the results of the regression controlling for year and district effects, which show a significant positive correlation between the experimental back-to-business policy and the upgrading of the district’s industrial structure by more than 5%; Column (4) of Table 3 shows that the coefficient of the impact of the policy of returning to the hometown to start a business on the level of the region’s industrial structure is 0.0244, which is significant at the level of 1%, holding the other factors unchanged. In conclusion, hypothesis H1 is confirmed.

**Table 3:** The impact of migrant laborers going back to their native towns to initiate their enterprises on the enhancement of industrial frameworks.

Industrial structure level	(1)	(2)	(3)	(4)
Pilot policy on entrepreneurship for migrant workers returning to their hometowns	0.0140** (0.0055)	0.0157*** (0.0058)	0.0163*** (0.0059)	0.0170*** (0.0058)
Level of economic development		-0 .0381 (0.0059)	0.0404*** (0.0062)	0.0507*** (0.0065)
Level of education			0.5050*** (0.1207)	0.4269*** (0.1158)
Level of communication			-0.0140 (0.0136)	-0.0173 (0.0132)
Level of social welfare				-0.0001 *** (0.0001)
Size of Government				0.0755*** (0.0163)
Constant	2.149*** (0.0002)	1.7681*** (0.0573)	1.6914*** (0.0597)	1.5829*** (0.0632)
Year fixed effects	YES	YES	YES	YES
County fixed effects	YES	YES	YES	YES
Observations	49,737	36,593	32,213	31,180
R^2^	0.8332	0.8613	0.8283	0.8418

Note

(1) *, **, and *** denote significance at 10%, 5%, and 1% significance levels, respectively; (2) robust standard errors are in parentheses

### 5.2 Parallel trend test

The success of the double difference method depends on the premise that the policy for returning to work in entrepreneurship aligns with the parallel trend hypothesis, implying that both the experimental and control groups should demonstrate a similar pattern of alteration due to the lack of a pilot policy for returning to work in entrepreneurship. Pertaining to the research concept proposed by Ma Qingshan and colleagues, this document develops a dynamic double difference model for parallel trends through the introduction of a policy dummy variable:

$$Upgrade_{i,\;t}=\alpha_{0}+\Sigma \alpha_{k}DID_{k,\;i,\;t}+\Sigma_{n}\alpha_{n}X_{i,\;t}+\mu_{i}+\delta_{t}+\varepsilon_{i,\;t}$$ (3)

**Fig 1:**
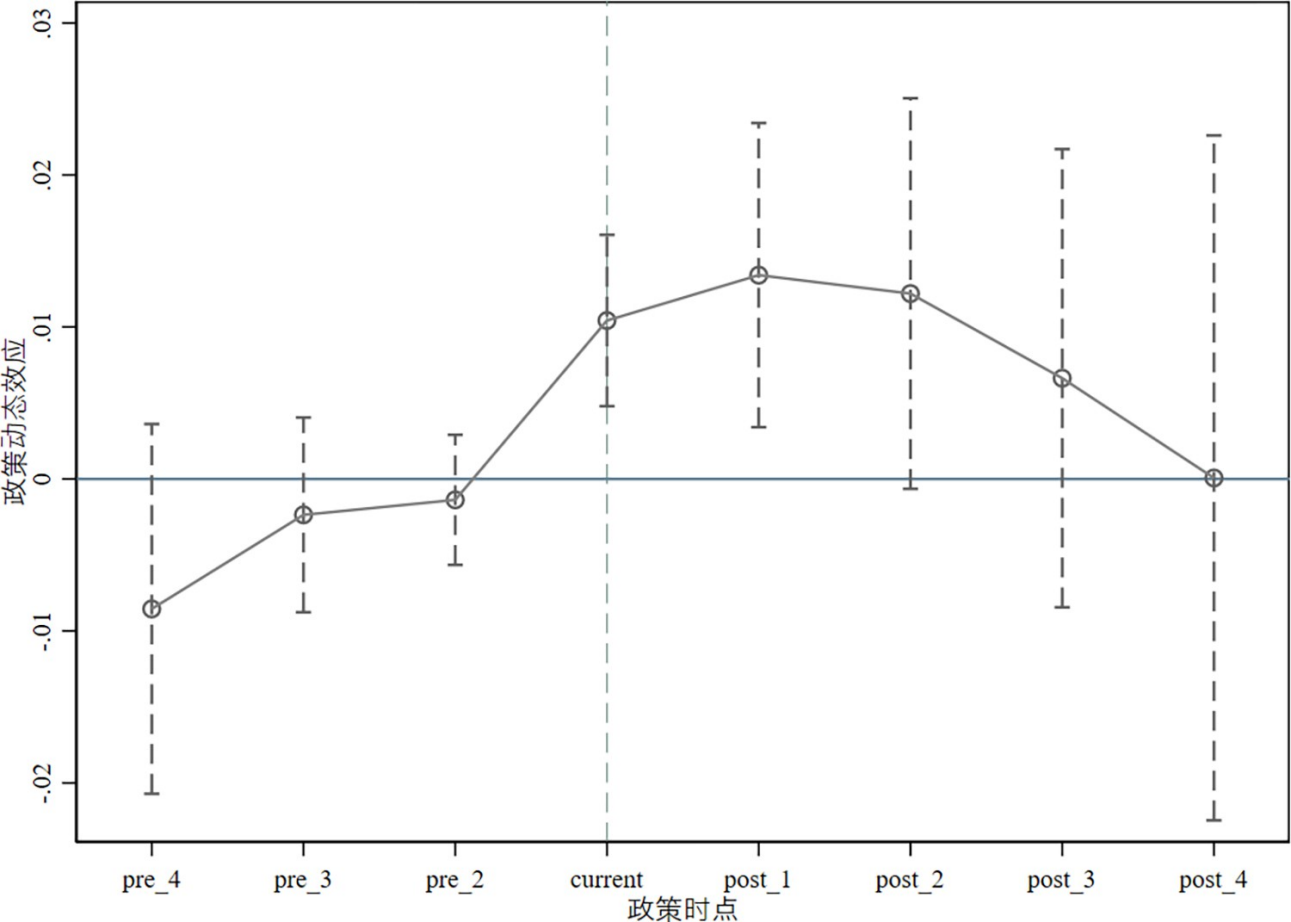
Parallel trend test.

*DID*_*k*, *i*, *t*_ indicates that it takes the value of 1 in the kth year of the approved pilot district and county of returning home to start business, otherwise it takes the value of 0, -4≤k≤4, k≠-1. The meaning of the rest of the variables’ symbols is consistent with Eq (1). Fig 1 shows that there was no significant difference between the control group and the experimental group before the implementation of the return-to-hometown entrepreneurship pilot policy, indicating that the sample meets the parallel trend assumption. The figure also shows that the industrial structure level of the control group and the experimental group has improved after the policy was implemented, indicating that the study sample meets the parallel trend assumption. The influence effect is no longer significant from the second year after the policy was implemented, which may be because the policy has certain limitations, and the influence is difficult to maintain.

### 5.3 Placebo test

**Fig 2:**
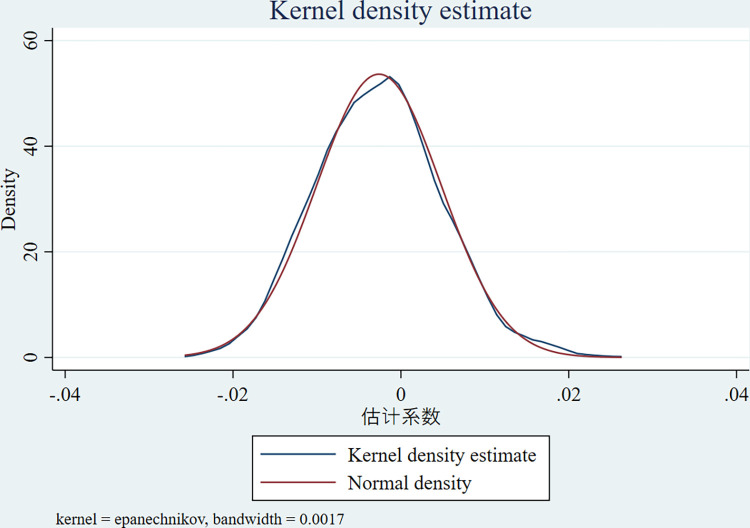
Placebo test results.

In order to avoid being affected by other unobserved variables, based on the study of LI et al. [33], the counties and districts in the original treatment group that set up the pilot of the policy of returning migrant workers to their hometowns for entrepreneurship are considered as the new control group; If n districts and counties are approved as the pilot in the next year t, keeping the time of the pilot of returning migrant workers to their hometowns for entrepreneurship unchanged, the districts and counties that did not set up the pilot of returning migrant workers to their hometowns for entrepreneurship are randomly sampled and the n treatment groups are obtained, and the model (4) of Fig 2 is re-estimated based on the new sample. Repeating the above process 1000 times, the mean value of the estimated coefficients is negative, while the estimated coefficients in model (4) of Fig 2 are positive, and this result indicates that the pilot policy of rural migrant workers returning to their hometowns to start their own businesses has obvious location-oriented characteristics. Advocating for the pilot policy within the local industrial framework remains largely unaffected by various random elements, underscoring the solidity of this primary finding.

### 5.4 Other robustness tests

First, the PSM-DID estimate. Although the dual difference method can identify the relative differences in industrial structure between pilot areas and non-pilot areas, there may still be selective bias in the observation of research data. According to the results from the Table 4 (1) column, the coefficients of the core explanatory variables for the pilot policy on returning home to start a business are not significantly different from those of Table 3, and the coefficients of the remaining control variables are in line with expectations, indicating that the benchmark regression results remain robust when selection bias is taken into account.

**Table 4:** Results of other robustness tests.

	PSM-DID estimate	(2) Changing the sample size	(3) correction of outliers	(4)Exclusion of other policies
Pilot policy on entrepreneurship for migrant workers returning to their hometowns (did)	0.0167*** (0.0061)	0.0099* (0.0059)	0.0172*** (0.0057)	0.0144*** (0.0051)
Control Variable	YES	YES	YES	YES
Year fixed effects	YES	YES	YES	YES
County fixed effects	YES	YES	YES	YES
Observations	30869	14,200	31,180	24,702
R^2^	0.5826	0.8866	0.8429	0.8735

Note

(1) *, **, and *** denote significance at 10%, 5%, and 1% significance levels, respectively

Second, change the sample size. For the reliability of the empirical results, this paper excludes the sample from the areas with relatively perfect infrastructure in county-level cities and districts, and only estimates the sample data of 1432 counties. After the readjustment of the whole sample, the pilot coefficient of returning home to start business is still significantly positive, which shows that returning home to start business of migrant workers has a positive impact on the level of the county industrial structure, and once again verified the reliability of this study.

Third, correction of outliers. In order to ensure the accuracy of the empirical results, this study controls for outliers that may interfere with the results. The traditional bilateral 1% shrinkage treatment lacks reliability, and this paper adopts the method of plotting box-and-line diagrams to rationalize the outliers. With this treatment, we obtain the estimation results as shown in column 4(3) of Table 4. The coefficient is significant at the 1% level at 13.4038 and the benchmark regression results are robust.

Fourth, other policy effects are excluded. During the research period of this paper, the state also issued the policies of e-commerce into rural demonstration counties and leisure agriculture and rural tourism demonstration counties, which are closely related to the research object of this paper Therefore, in order to exclude the influence of other policies on the empirical results, this paper adopts the antecedent factors, such as whether it is an e-commerce into rural areas demonstration county and whether it is a leisure agriculture and rural tourism demonstration county, as the proxy variables and interacts with the time trend term interacted and added to the benchmark regression. The results show that the regression results are significantly positive at the 1% level after controlling for the two model county policies. The reliability and robustness of the estimation results are verified.

### 5.5 Heterogeneity analysis

This study will classify the whole sample into eastern, central and western cities according to the regional division standard of the National Bureau of Statistics, in order to verify the difference of the impact of the pilot policy of returning to the hometown to start a business on the level of industrial structure of counties in different regions, so as to facilitate further research and analysis. Construct the dummy variable of eastern cities (eastprovince), the dummy variable of central and western cities (middlewestprovince) respectively, and after cross-multiplying with did and set the following model:

$$Upgrade_{i,\;t}=\alpha_{0}+\alpha_{1}DID_{i,\;t}\times area+\alpha_{2}X_{i,\;t}+\mu_{i}+\delta_{t}+\varepsilon_{i,\;t}$$ (4)

According to the results in Table 5, it can be seen that the regression coefficients of the pilot policy of returning home to start a business in the central and western regions show a positive correlation at the 1% level, indicating that the policy plays a positive role in promoting the upgrading of the industrial structure of the counties in the region. In contrast, the variables of the pilot policy of returning home to start a business in the eastern region failed the significance test, indicating that the return of workers to start a business does not play a positive role in the region. This is because the eastern region has a developed economy, a relatively solid industrial base and better support facilities. Comparatively, the industrial structure of the central and western regions is at a lower level, and the lack of production factors such as material resources and highly skilled personnel limits the upgrading of the industrial structure. Migrant workers returning to the countryside to start businesses will be useful to explore the advantages of local resources, promote the return of production factors, encourage more surplus labor to start businesses, and mitigate brain drain. At the same time, the national strategy for the rise of central China, the western development strategy and other policies also provide support for the optimization and upgrading of the county’s industrial structure.

**Table 5:** Results of heterogeneity analysis.

Explanatory variables	Level of industrial structure			
Eastprovince*DID	0.0076 (0.0091)			
Middleweastprovince* DID		0.0190*** (0.0067)		
old*DID			0.0250*** (0.0070)	
unold*DID				0.0054 (0.0089)
Control Variables	YES	YES	YES	YES
Year fixed effects	YES	YES	YES	YES
County fixed effects	YES	YES	YES	YES
Number of observations	31180	31180	31180	31180
R-squared	0.8416	0.8418	0.8418	0.8416

Note

(1) *, **, and *** denote significance at 10%, 5%, and 1% significance levels, respectively; (2) robust standard errors are in parentheses

The degree of aging may also be the reason for the different effects of the implementation of the pilot policy of rural migrant workers returning to their hometowns for entrepreneurship on the upgrading of the industrial structure. Internationally, it is considered that counties where the proportion of people aged 65 and above in the total population reaches 14% have entered a deeply aging society. In this paper, the whole sample is divided into two groups of deep aging and non-deep aging, and according to the above method, we construct the dummy variable of deep aging (old) and non-deep aging (unold), respectively, and construct the regression model after cross-multiplying with DID. From the regression results in Table 5, it can be seen that in deep aging counties, the implementation of the pilot policy of returning to entrepreneurship has a significant positive effect, and its coefficient estimate shows an obvious positive effect, indicating that the pilot policy of returning to entrepreneurship in deep aging counties plays a significant role in promoting the upgrading of industrial structure, This is probably because the non-deeply aging counties are likely to be relatively young and their industrial structure may be more skewed toward traditional industries, while the policy of migrant workers returning home to start businesses may be more related to emerging industries and services. Over a brief period, while these policies might affect conventional industries, they don’t always lead to the enhancement of the local industrial framework. The validation of Hypothesis H2 has been achieved.

## 6 Analysis of mechanisms

The preceding section’s theoretical examination reveals that the primary drivers behind the enhancement of the county’s industrial framework could be the innovation promotion effect and the market push effect. According to the estimation results shown in Table 6 (1), the coefficients of the pilot policy variable of returning to hometown entrepreneurship are significantly positive at the 5% level, indicating that the pilot policy has a certain promotion effect on the level of innovation and technology. (Column (2) introduces the level of market consumption as an explanatory variable, and the results show that the pilot policy is significantly positive at the 5% level, indicating that the policy of returning to the hometown entrepreneurship can promote the level of market consumption. From the previous discussion, we can clearly see that this experimental project to encourage workers to return to their hometowns for entrepreneurship has brought about various impacts. First, the policy introduces new technologies, promotes market development, improves product quality and service level, promotes the optimization of traditional industries, and meets the needs of the public. Second, the policy has raised local consumption levels, prompted producers to adjust their scale and product types, and promoted supply-side industrial upgrading. Therefore, hypotheses H3 and H4 are verified.

**Table 6:** Mechanism tests.

	Level of innovative technology	Level of Market Consumption
Pilot policy on entrepreneurship for migrant workers returning to their hometowns (did)	0.7643** (0.3457)	0.0456** (0.0190)
Control Variable	YES	YES
Year fixed effects	YES	YES
County fixed effects	YES	YES
Observations	8,210	30,537
R^2^	0.8522	0.9696

Note

(1) *, **, and *** denote significance at 10%, 5%, and 1% significance levels, respectively; (2) robust standard errors are in parentheses

## 7 Conclusions and limitations

### 7.1 Conclusions and insights

This study empirically examines the link between localization policies and strengthening the industrial framework of the county through a quasi-natural experiment, namely the policy of returning home for entrepreneurship. Based on the panel data of 1997 counties across China from 2000 to 2021, using a multi-temporal double-difference model, it shows that the pilot policy of returning home to start a business significantly promotes the upgrading of county industrial structure. Further analysis indicates regional differences in the impact. Industrial upgrades are more significant in the central and western regions, especially in aging areas. Mechanism tests show that the pilot policy for returning entrepreneurship promotes industrial upgrading through innovation and market effects.

Therefore, this paper makes the following policy suggestions:

First, improve the policy of entrepreneurship for hometown return. To give more effective play to the functions of the government, promote the formation of a working pattern of cross-sectoral coordination, upward and downward linkage and policy synergy, and guide the entrepreneurship of returning and local migrant workers to achieve a higher level of development. Organizational safeguards will be strengthened to ensure the effective implementation of policies and measures on entrepreneurship for returnees and local residents. Deepen the reform of "releasing administrative services" and optimize the business environment to support the entrepreneurship of returnees and local residents, and strengthen financial and fiscal support to reduce the cost of production and operation of enterprises.

Second, attention should be paid to the problem of population aging, and differentiated policies should be implemented in the eastern, central and western regions. The government should implement differentiated policies in the eastern, central and western regions. For the eastern region, it can provide more entrepreneurial support policies, such as financial subsidies and tax incentives, to motivate young people to return to their hometowns to start their own businesses, increase the regional fertility rate, and promote industrial structure upgrading. As for the central and western regions, the impact factors of population aging should be fully considered, and appropriate measures should be taken to alleviate the aging pressure, while more policy support and resources should be provided to encourage entrepreneurs to return to their hometowns, so as to promote the transformation and upgrading of the local industrial structure, and enhance the competitiveness of the regional economy and its development potential. Taking into account the characteristics and needs of each region, the implementation of differentiated population policies will be more conducive to solving the problem of population aging and promoting the healthy development of regional economies.

Third, optimize the consumption structure and promote technological upgrading. In promoting the internal circulation of the economy, the focus is on cultivating emerging industries that align with local resources and market demand, guided by consumer demand. It supports the technological transformation and upgrading of traditional industries to enhance competitiveness and value-added. Enhancing the availability of superior products and services, upgrading the social security framework, and broadening the market for rural consumers are key to boosting the spending of rural inhabitants and fostering the swift growth of the rural economy.

### 7.2 Limitations and future research directions

#### 7.2.1 Limitations

*1*. *Static nature of policy assessment*. In assessing the policy of returning to the countryside to start a business, this paper focuses mainly on the immediate impact of the policy implementation and lacks a dynamic assessment of the effect of the policy over time. In the real situation, the effect of the policy may change with the change of the external environment and the increase of the depth of policy implementation. For example, the policy may have a significant driving effect on the upgrading of county industrial structure at the initial stage, but this driving effect may weaken or new problems may emerge with the passage of time. Future research can adopt a dynamic tracking approach to examine the long-term impact of the return-to-work policy on the county economy and social structure at different stages.

*2*. * Dependence on macroeconomic indicators*. This paper relies more on macroeconomic indicators, such as GDP growth rate and employment rate, in assessing the impact of the return-to-land entrepreneurship policy, which, while providing an overall overview of economic activities, may not fully capture the specific impact of the policy on individual entrepreneurs and micro and small economies. In addition, macroeconomic indicators may mask structural changes and localised regional differences that are critical to a full understanding of the impact of the return-to-work policy. Future research could consider combining microeconomic data, such as household income, consumption patterns, and business survival rates, to supplement macroeconomic indicators.

*3*. * Limitations of the analysis method*. This paper mainly adopts quantitative analysis methods, which may fail to fully reveal the internal mechanism of the impact of the return-to-work policy, although it provides strong statistical evidence. Qualitative analyses, such as in-depth interviews and case studies, can provide richer insights, but this aspect is not fully developed in this paper. In addition, quantitative analyses may not fully capture the unintended consequences of policy implementation, such as environmental impacts, potential changes in social inequality, etc. Future research could use mixed-method studies that combine quantitative and qualitative data to gain a more comprehensive understanding.

#### 7.2.2 Future research outlook

*1*. *Implementation of dynamic policy evaluation*. Future research could implement dynamic policy evaluation, which involves continuous monitoring and evaluation of the effects of return-to-work policies. By establishing a long-term tracking research framework, data can be collected and analyzed at different points in time after the implementation of the policy, thus revealing the evolution of policy effects over time. This dynamic assessment method can not only help researchers understand the short- and long-term effects of the policy, but also identify new problems and challenges that may arise during the implementation of the policy, providing a basis for policy adjustment and optimization. In addition, dynamic assessment can also take into account changes in the policy environment, such as the impact of economic cycle fluctuations, technological progress, demographic changes and other factors on policy effects.

*2.* *Incorporating microeconomic data*. Future research could analyze microeconomic data in conjunction with microeconomic data in order to more accurately measure the impact of return-to-work entrepreneurship policies at the individual and community level. This would include collecting and analyzing data on the financial status of returnee entrepreneurs, the profitability of their businesses, their job creation capacity, and their impact on local supply chains and markets. Through micro-level analyses, research can reveal how policies affect different sizes and types of businesses, and how these impacts are transmitted and diffused in communities. In addition, microdata can help identify possible inequalities in policy implementation and inform the development of more equitable and effective policies.

*3.* *Incorporating qualitative research methods*. Future studies could adopt qualitative research methods, such as semi-structured interviews or focus groups, in order to explore in depth, the personal experiences and perceptions of returning entrepreneurs. This will help to complement quantitative analyses and provide a more comprehensive policy assessment. For example, interviews can be used to understand the main challenges faced by returning entrepreneurs, the actual effects of policy support, and their expectations and suggestions for policy. In addition, qualitative research can reveal the unintended consequences of policy implementation, such as the impact on the local social structure, cultural traditions and environment. By combining quantitative and qualitative research, future studies can provide deeper and richer policy analyses.

## References

[1] Neumark D.; Simpson H. Place-based policies. In *Handbook of regional and urban economics*, Elsevier: 2015; Vol. 5, pp 1197–1287.

[2] Duranton G.; Venables A.J. Place-based policies for development; National Bureau of Economic Research: 2018; p.

[3] Wang X.; Feng Y. The effects of National High-tech Industrial Development Zones on economic development and environmental pollution in China during 2003–2018. *Environ Sci Pollut R* 2021, 28, 1097–1107. 10.1007/s11356-020-10553-1 32829438

[4] Liu J.; Li N.; Du X. Did place-based industrial policy promote regional economic growth?—-Evidence from China. *Plos One* 2023, 18, e283688. doi: 10.1371/journal.pone.0283688 37014899 PMC10072470

[5] Wang Z.; Yang Y.; Wei Y. Has the Construction of National High-Tech Zones Promoted Regional Economic Growth?—Empirical Research from Prefecture-Level Cities in China. *Sustainability-Basel* 2022, 14, 6349.

[6] Li Y.; Zhang J.; Yang X.; Wang W.; Wu H.; Ran Q.; et al. The impact of innovative city construction on ecological efficiency: A quasi-natural experiment from China. *Sustain Prod Consump* 2021, 28, 1724–1735.

[7] Yang J.; Xiong G.; Shi D. Innovation and sustainable: can innovative city improve energy efficiency? *Sustain Cities Soc* 2022, 80, 103761.

[8] Li X.; Tang J.; Huang J. Place-based policy upgrading, business environment, and urban innovation: Evidence from high-tech zones in China. *Int Rev Financ Anal* 2023, 86, 102545.

[9] Zhou L.; Du L. Improving the sustainable development of firms: Evidence from the environmental constraint target policy in China. *Sustain Dev* 2024, 32, 1217–1225.

[10] Peng H.; Ling K.; Zhang Y. The carbon emission reduction effect of digital infrastructure development: Evidence from the broadband China policy. *J Clean Prod* 2024, 434, 140060.

[11] Chien S. The isomorphism of local development policy: A case study of the formation and transformation of national development zones in post-Mao Jiangsu, China. *Urban Stud* 2008, 45, 273–294.

[12] Puga D. European regional policies in light of recent location theories. *J Econ Geogr* 2002, 2, 373–406.

[13] Vanhoudt P.; Mathä T.; Schmid B. How productive are capital investments in Europe? *Eib Papers* 2000, 5, 81–106.

[14] Dall’Erba S.; Le Gallo J. Regional convergence and the impact of European structural funds over 1989–1999: A spatial econometric analysis. *Pap Reg Sci* 2008, 87, 219–245.

[15] Ulltveit-Moe K.H. *Live and Let Die*: *Industrial Policy in a Globalised World*. Citeseer: 2008; p.

[16] Collier P. *The bottom billion*: *Why the poorest countries are failing and what can be done about it*. Oxford University Press, USA: 2008; p.

[17] Rodríguez Pose A.; Arbix G. Strategies of waste: bidding wars in the Brazilian automobile sector. *Int J Urban Regional* 2001, 25, 134–154.

[18] Callais J.T.; Peng L. The impact of place-based policy: evidence from a multiple synthetic control analysis of the northeastern revitalization program in China. *J Asia Pac Econ* 2022, 1–20.

[19] Renski H. New firm entry, survival, and growth in the United States: A comparison of urban, suburban, and rural areas. *J Am Plann Assoc* 2008, 75, 60–77.

[20] He C.; Lu J.; Qian H. Entrepreneurship in China. *Small Bus Econ* 2019, 52, 563–572.

[21] Bosma N.; Sternberg R. Entrepreneurship as an urban event? Empirical evidence from European cities. In *Entrepreneurship in a Regional Context*, Routledge: 2017; pp 78–95.

[22] Mohamed M.; Abdul-Talib A. Push–pull factors influencing international return migration intentions: a systematic literature review. *Journal of Enterprising Communities*: *People and Places in the Global Economy* 2020, 14, 231–246.

[23] Baumol W.J. Entrepreneurship in economic theory. *The American Economic Review* 1968, 58, 64–71.

[24] Krichevskiy D. A consumption-based measure of the monetary rewards to entrepreneurship. *J Entrep Public Poli* 2014, 3, 49–71.

[25] Hanlon W.W. Necessity is the mother of invention: Input supplies and Directed Technical Change. *Econometrica* 2015, 83, 67–100.

[26] King R.G.; Levine R. Finance, entrepreneurship and growth. *J Monetary Econ* 1993, 32, 513–542.

[27] Baumol W.J. Macroeconomics of unbalanced growth: the anatomy of urban crisis. *The American Economic Review* 1967, 57, 415–426.

[28] Audretsch D.B.; Keilbach M. Resolving the knowledge paradox: knowledge-spillover entrepreneurship and economic growth. *Res Policy* 2008, 37, 1697–1705.

[29] Feng Y.; Lee C.; Peng D. Does regional integration improve economic resilience? Evidence from urban agglomerations in China. *Sustain Cities Soc* 2023, 88, 104273. doi: 10.1016/j.scs.2022.104273

[30] Baker T.; Welter F. *Contextualizing entrepreneurship theory*. 2020; p.

[31] Gao Y.D.; Zhang W.G.; Yang Q. The factors influencing of industrial structure upgrade in China. *Econ Geogr* 2015, 35, 96–101.

[32] Ruan J.Q., Yang Q.M., Ye W.W., Zhang Y.W. Innovation and entrepreneurship development in rural China: index construction and measurement analysis[J]. *Economy and Management*,2024(5):9–18.

[33] Li P.; Lu Y.; Wang J. Does flattening government improve economic performance? Evidence from China. *J Dev Econ* 2016, 123, 18–37.

